# Altered Long- and Short-Range Functional Connectivity Density in Healthy Subjects After Sleep Deprivations

**DOI:** 10.3389/fneur.2018.00546

**Published:** 2018-07-16

**Authors:** Dan Kong, Run Liu, Lixiao Song, Jiyong Zheng, Jiandong Zhang, Wei Chen

**Affiliations:** ^1^Department of Medical Imaging, The Affiliated Huai'an No. 1 People's Hospital of Nanjing Medical University, Huai'an, China; ^2^Department of Radiology, The Affiliated Xi'an Central Hospital of Xi'an Jiaotong University, Xi'an, China; ^3^Department of Hematology, The Affiliated Huai'an No. 1 People's Hospital of Nanjing Medical University, Huai'an, China; ^4^Department of Interventional Radiology, The Affiliated Huai'an No. 1 People's Hospital of Nanjing Medical University, Huai'an, China

**Keywords:** sleep deprivation, functional connectivity density, receiver operating characteristic, sensorimotor, short-range, long-range

## Abstract

**Objective:** To investigate the brain functional organization induced by sleep deprivation (SD) using functional connectivity density (FCD) analysis.

**Methods:** Twenty healthy subjects (12 female, 8 male; mean age, 20.6 ± 1.9 years) participated a 24 h sleep deprivation (SD) design. All subjects underwent the MRI scan and attention network test twice, once during rested wakefulness (RW) status, and the other was after 24 h acute SD. FCD was divided into the shortFCD and longFCD. Receiver operating characteristic (ROC) curve was used to evaluate the discriminating ability of those FCD differences in brain areas during the SD status from the RW status, while Pearson correlations was used to evaluate the relationships between those differences and behavioral performances.

**Results:** Subjects at SD status exhibited lower accuracy rate and longer reaction time relative to RW status. Compared with RW, SD had a significant decreased shortFCD in the left cerebellum posterior lobe, right cerebellum anterior lobe, and right orbitofrontal cortex, and increased shortFCD in the left occipital gyrus, bilateral thalamus, right paracentral lobule, bilateral precentral gyrus, and bilateral postcentral gyrus. Compared with RW, SD had a significant increased longFCD in the right precentral gyrus, bilateral postcentral gyrus, and right visuospatial network, and decreased longFCD in the default mode network. The area under the curve values of those specific FCD differences in brain areas were (mean ± std, 0.933 ± 0.035; 0.863~0.977). Further ROC curve analysis demonstrated that the FCD differences in those brain areas alone discriminated the SD status from the RW status with high degree of sensitivities (89.19 ± 6%; 81.3~100%) and specificities (89.15 ± 6.87%; 75~100%). Reaction time showed a negative correlation with the right orbitofrontal cortex (*r* = −0.48, *p* = 0.032), and accuracy rate demonstrated a positive correlation with the right default mode network (*r* = 0.573, *p* = 0.008).

**Conclusions:** The longFCD and shortFCD analysis might be potential indicator biomarkers to locate the underlying altered intrinsic brain functional organization disturbed by SD. SD sustains the cognitive performance by the decreased high-order cognition related areas and the arousal and sensorimotor related areas.

## Introduction

Sleep deprivation, widespread in current society, can be caused by environmental factors or personal reasons. It generally has a deleterious effect on emotional regulation, memory, attention, and executive control function (1–5), and even metabolic, physiological, psychological, and/or behavioral reactivity with a greater risk of being multiorgan and multisystem dysfunction (6–9). Recently, several studies have demonstrated structural and functional changes in the frontal cortex, parietal cortex, and temporal cortex in individuals after acute SD (1, 6, 10–21); however, the neurologic mechanism of acute SD has not been fully studied.

Resting-state functional MRI (rfMRI) can combine the functional images and structural images without exposure to radioactive tracers, which makes the rfMRI suitable for mechanism and pathophysiology exploration in several diseases (1). The advance of rfMRI can help us non-invasively explore the functional organization in the human brain thus better characterize the changes of regional neuronal spontaneous brain activity and intrinsic connectivity patterns to understand the underlying neural basis of neuropsychiatric disorders.

Seed-based functional connectivity studies have revealed abnormal connectivity patterns in individuals with insufficient sleep in brain regions related to emotion and cognition (13, 18, 21–26); however, the seed-based functional connectivity analysis provides limited information about the relationships between the time series of a given seed point area and the time series of other areas in a whole brain network (27, 28). Voxel-based functional connectivity density (FCD) was used to identify the distribution of hubs in the human brain (29). In contrast to the seed-based functional connectivity analysis, the FCD analysis, similar to the degree centrality analysis, provides an opportunity for unbiased searches abnormalities within the whole brain without the need for a prior definition of regions of interest (27). The FCD can be divided into the short-range FCD and long-range FCD on the basis of the neighboring relationships between brain voxels (30). Recently, the FCD analysis has been widely applied to the exploration of the neurophysiological basis of several diseases (31–34), and reveals extra information which cannot be provided by the seed-based functional connectivity analysis. In this framework, in the present study we utilized the potential indicators of shortFCD and longFCD approaches to characterize the changes of intrinsic functional connectivity strength after acute SD status relative to rested wakefulness (RW) status, and further explore the potential neurobiological mechanisms of SD.

## Materials and methods

### Subjects

Twenty healthy subjects (12 female, 8 male; mean age, 20.6 ± 1.9 years; mean education, 14.5 ± 1.19 years) participated in a 24 h SD design experiment. All subjects met the following criteria, as in previous studies (1, 6):

- Right-handed
- Good sleep habits without any symptoms of sleep disorders [such as difficulties in sleep onset (> 30 min) and/or maintaining sleep]
- Pittsburgh sleep quality index score < 5
- No consumption of any nicotine, hypnotic, or psychoactive medications, diet pills, alcohol, and caffeine for ≥ 3 months prior and during to the current study
- Regular dietary habit with moderate weight and body shape
- No foreign implants, inborn, and acquired diseases

Each of the subjects underwent the MRI scan twice; once during RW status, and the other after 24 h' acute SD. The acute SD process started at 19:00 on the first day and lasted until 07:00 in the second day. The food and water were provided during the SD procedure. The temperature of the room was maintained between 23°C and 27°C. The team took turns to monitor and make sure that the participants did not fall asleep using video monitors. This study was approved by the Medical Research Ethical Committee of The Affiliated Huai'an No. 1 People's Hospital of Nanjing Medical University in accordance with the Declaration of Helsinki. All volunteers participated voluntarily and were informed of the purposes, methods, and potential risks of this study, and signed an informed consent form.

### MRI

The MRI examination was performed, via acquisition, on a clinical 3T MRI scanner (SIEMENS Trio, Erlangen, Siemens, Germany) with a standard eight-channel head coil using a 12-channel array coil. First, we acquired a high-resolution 3D anatomical images with 176 T1-weighted images in a sagittal orientation: repetition time = 1950 ms, gap = 0 mm, echo time = 2.3 ms, thickness = 1 mm, acquisition matrix = 248 × 256, flip angle = 9°, field of view = 244 × 252 mm. Second, we also acquired 240 functional images using a single-shot Gradient-Recalled Echo-Planar Imaging pulse sequence (repetition time = 3000 ms, gap = 0.5 mm, echo time = 25 ms, thickness = 5.0 mm, flip angle = 90°, acquisition matrix = 32 × 32, field of view = 210 × 210 mm).

### Attention network test (ANT)

Before the MRI scan, all volunteers underwent an attention network test (ANT) (1, 12, 35, 36). The ANT contained three cue conditions (no cue, center cue, spatial cue) and two target conditions (congruent and incongruent). The visual stimuli consisted of a row of five horizontal black arrows pointing leftward or rightward with the target arrow in the center. The participants responded to the direction of the central arrow by pressing the left or right buttons of the computer mouse. The task measured alerting, orienting, and conflict effects by calculating the difference between the response time and the presentation time under three different cue conditions. The accuracy rate using corrected recognition, reaction time using only trials with correct responses, and lapse rate using missing recognition, were calculated.

### Data analysis

First, the first 10 time points of the functional images were deleted, due to the possible instability of the initial MRI signal. The remaining data was analyzed by Data Processing & Analysis for Brain Imaging (DPABI 2.1, http://rfmri.org/DPABI) toolbox based on MATLAB2010a (Mathworks, Natick, MA, USA). The data preprocessing contained the following steps: including the format transformation, slice timing, head motion correction spatial normalization to the Montreal Neurological Institute (MNI) space, and smooth. The data of participants with > 1.5 mm maximum translation in x, y, or z directions and >1.5° degree of motion rotation were removed. Based on the recent work showing that higher-order models benefit from the removal of head motion effects (37, 38), after the head motion correction, The functional images were re-sampled at a resolution of 3 × 3 × 3 mm^3^ during the spatial normalization. Linear regression was applied to remove the effects of spurious covariates, including the Friston 24 head motion parameters, global mean signal, white matter and cerebrospinal fluid signal. Next, the functional images were entered into temporally bandpass filtered (0.01–0.1 Hz) and linearly detrended.

### Calculation of long FCD and shortFCD calculation maps

The local and global FCD maps for each individual were calculated in a gray matter (GM) mask. The number of functional connections of a given voxel was considered as a degree of a node in a binary graph. First, we defined the functional connectivity between a given voxel with each of other voxels in the whole brain with a correlation threshold of *r* > 0.25 (39). In the present study, we adopted the threshold of *r* = 0.3 to calculate the FCD maps. Second, the longFCD and shortFCD were defined based on the neighborhood strategy. We defined the voxels with a correlation threshold of *r* > 0.25 inside their neighborhood (radius sphere ≤ 6 mm) as shortFCD, and defined the voxels with a correlation threshold of *r* > 0.25 outside their neighborhood (radius sphere > 6 mm) as long FCD. Next, the shortFCD and longFCD maps of each subject were divided by the mean value so as to convert to Z scores to improve the normality. Finally, the shortFCD and longFCD maps underwent spatial smoothing with a Gaussian kernel of 6 × 6 × 6 mm^3^ full-width at half-maximum using SPM8. The detailed procedure of the shortFCD and longFCD is given in a previous study (29).

### Statistical analysis

Data was presented as mean ± standard deviation (mean ± std). Pair *t*-tests were used for demographic factors (age, years of education, and ANT findings). *p* < 0.05 was considered as significant.

Pair *t*-tests were used to investigate the FCD differences in regional brain areas of the subjects during the acute SD status relative to the RW status. AlphaSim correction (threshold of individual voxel of *p* < 0.05 and cluster level of *p* < 0.05) was used to determine the statistical differences.

We used the receiver operating characteristic (ROC) curve to investigate the ability of those binarized FCD differences in regional brain areas to distinguish the SD status from the RW status, and we used Pearson correlations to evaluate the relationships between those binarized FCD differences in brain areas and ANT during the SD status. The statistical threshold was set at *P* < 0.05.

## Results

### Ant findings

**Figure 1:**
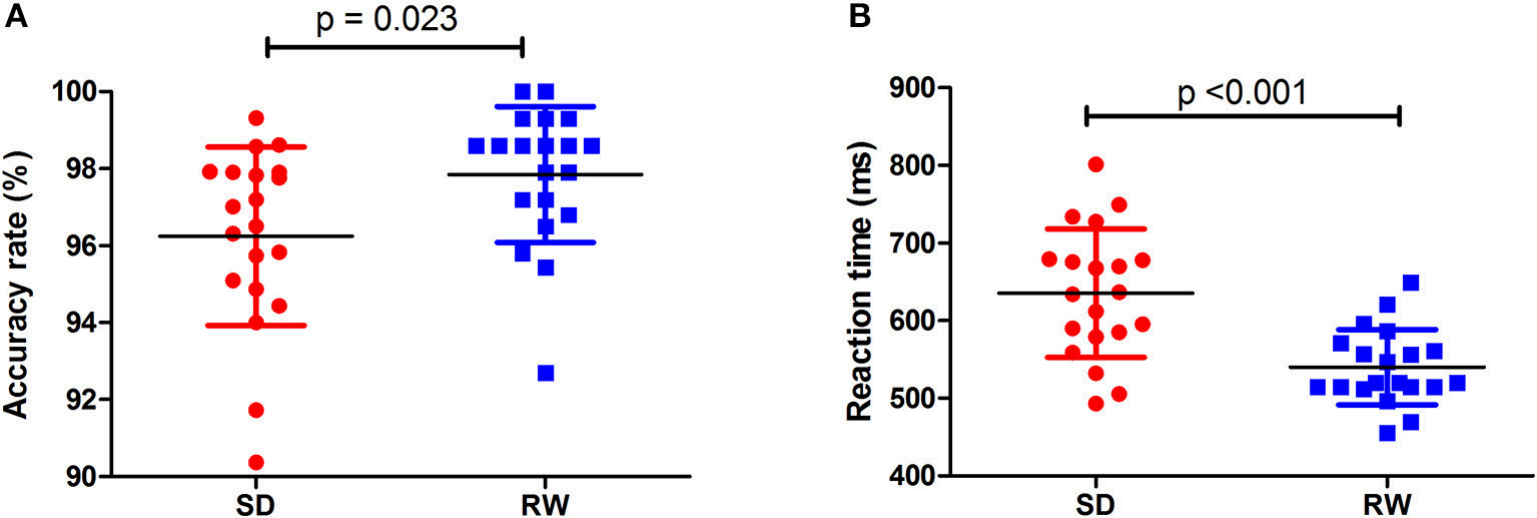
Behavioral findings of ANT. The accuracy rate and reaction time of RW group **(A)** and SD group **(B)**, respectively. Data is presented as mean ± standard error. ANT, Attention network test; SD, Sleep deprivation; RW, Rested wakefulness.

Individuals at acute SD status showed a lower accuracy rate (acute SD = 96.25 ± 2.32%, RW = 97.85 ± 1.77%; *t* = −2.482, *p* = 0.023; Figure 1A) and a longer reaction time (acute SD = 635.27 ms ± 82.68 ms; RW = 540.01 ± 48.37 ms; *t* = 5.013, *p* < 0.001; Figure 1B) during the ANT relative to the individuals at RW status.

### FCD differences between-groups

**Figure 2:**
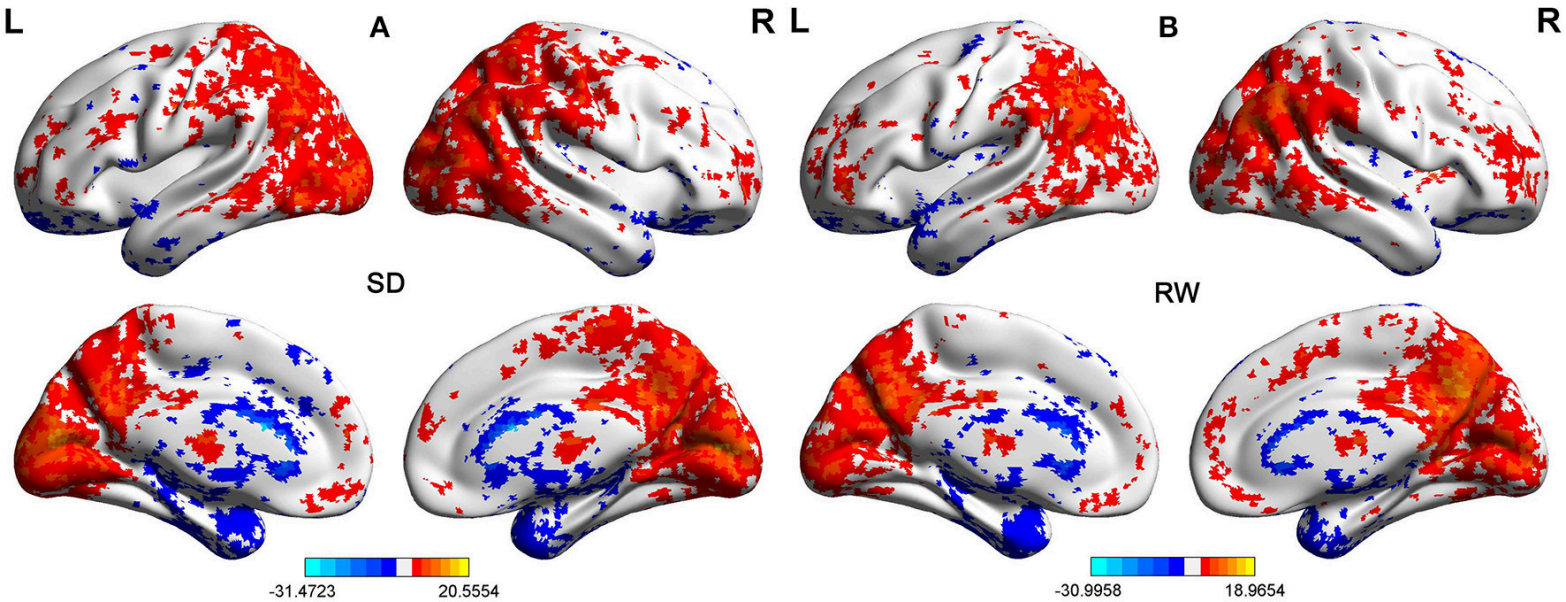
One sample *t*-test differences of SD and RW in Binarized shortFCD. The Binarized shortFCD maps in the SD group **(A)** and the RW group **(B)**, respectively. These maps are the results of the within-groups using one-sample *t*-tests, corrected by FDR. L, left; R, right; SD, sleep deprivation; RW, rested wakefulness; shortFCD, short-range functional connectivity density; FDR, false discovery rate.

**Figure 3:**
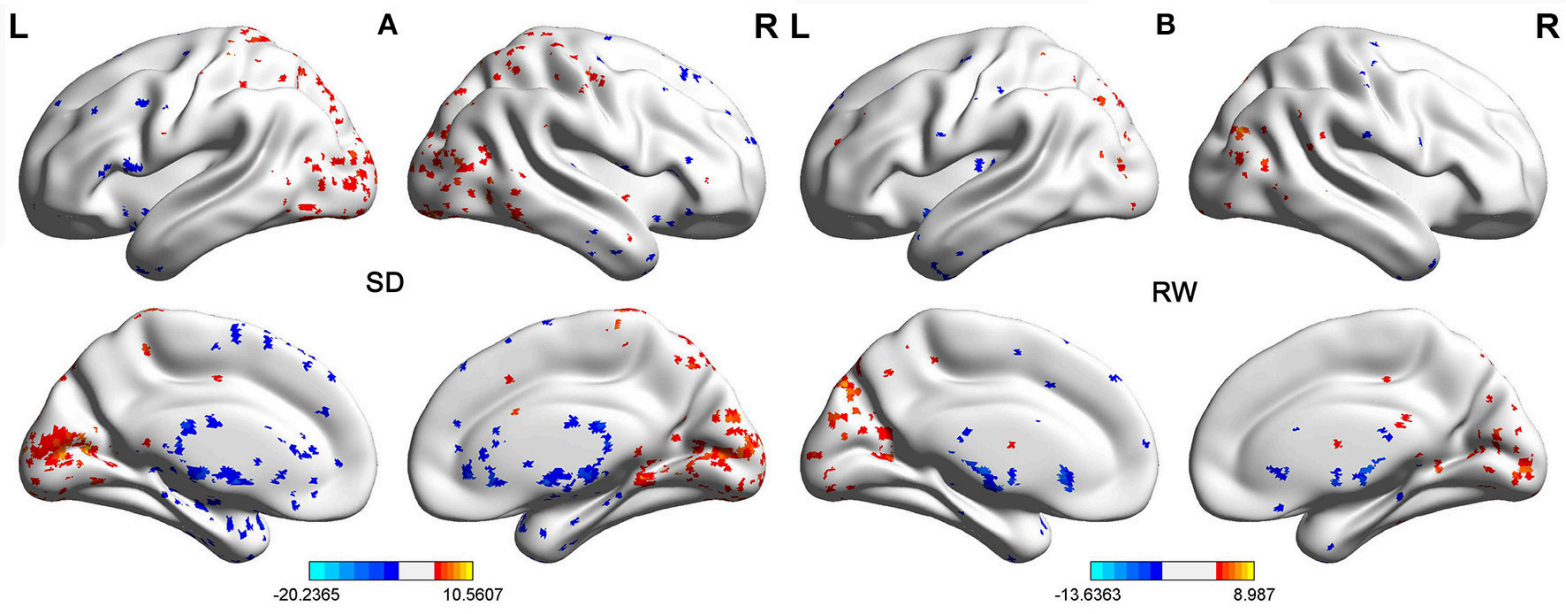
One sample *t*-test differences of SD and RW in Binarized longFCD. The Binarized longFCD maps in the SD group **(A)** and the RW group **(B)**, respectively. These maps are the results of the within-groups using one-sample *t*-tests, corrected by FDR. L, left; R, right; SD, sleep deprivation; RW, rested wakefulness; longFCD, long-range functional connectivity density; FDR, false discovery rate.

First, we performed one-sample *t*-test to explore the FCD differences at within-group level for each group. Figure 2 shows the shortFCD maps in the SD group (Figure 2A) and RW group (Figure 2B), respectively. Figure 3 shows the longFCD maps in the SD group (Figure 3A) and RW group (Figure 3B), respectively. The covered differences in brain areas both in binarized shortFCD and in binarized longFCD were larger in the SD group than that of RW group.

**Figure 4:**
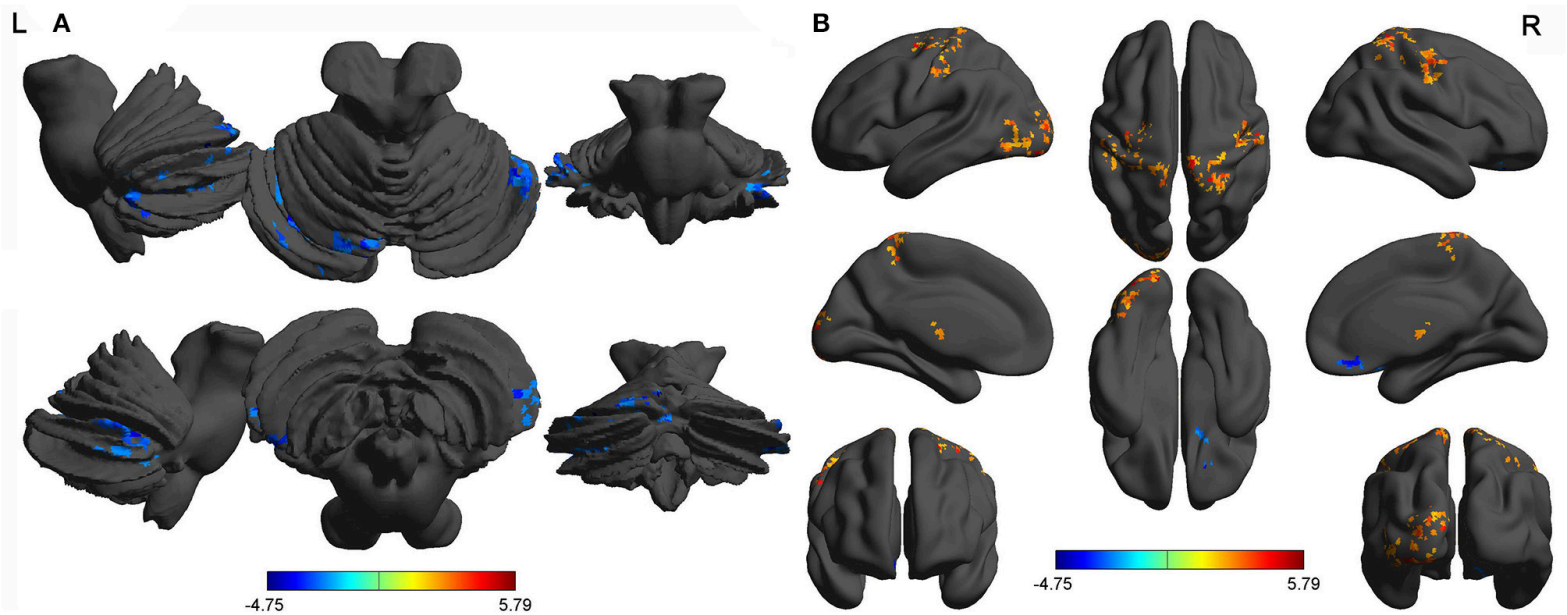
Binarized shortFCD differences between SD and RW. The color in the map represents the differences. The blue signifies decreased binarized shortFCD in brain areas **(A)**, and the red color signifies increased binarized shortFCD in brain areas **(B)**. shortFCD, short-range functional connectivity density; SD, Sleep deprivation; RW, Rested wakefulness; L, left; R, right.

**Figure 5:**
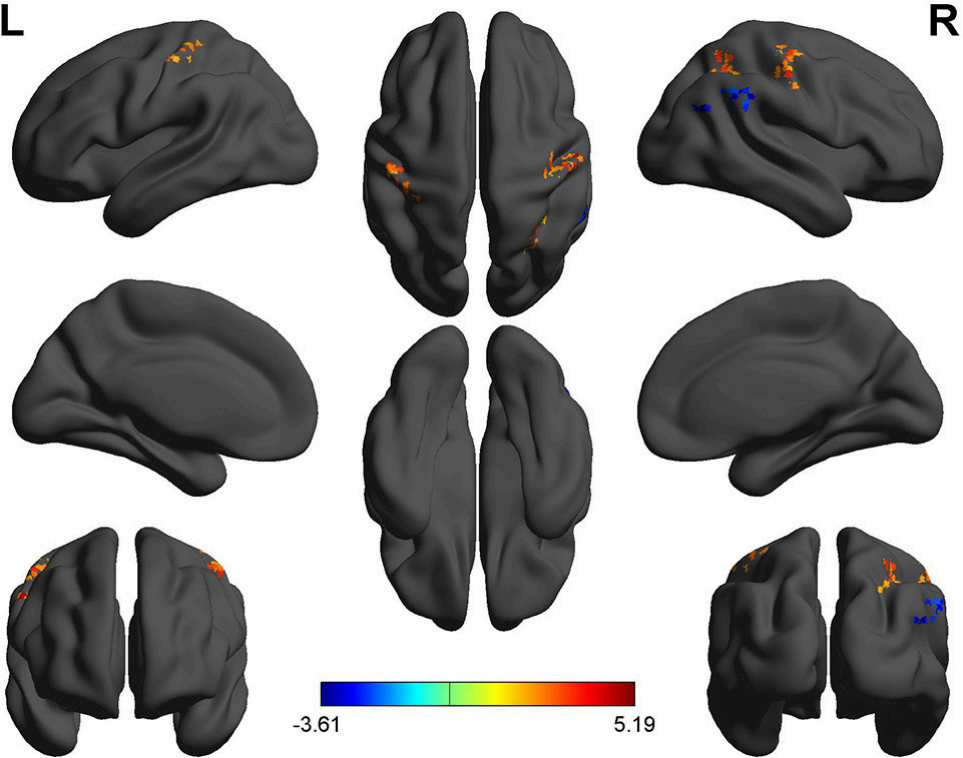
Binarized longFCD differences between SD and RW. The color in the map represents the differences. The red color signifies increased binarized longFCD in brain areas, and the blue signifies decreased binarized longFCD in brain areas. longFCD, long-range functional connectivity density; SD, Sleep deprivation; RW, Rested wakefulness; L, left; R, right.

Second, we performed pair t-tests to explore the FCD differences between-groups. Compared with RW, acute SD had significant decreased binarized shortFCD areas in the left cerebellum posterior lobe, right cerebellum anterior lobe (Figure 4A) and right inferior frontal gyrus (orbitofrontal cortex), and increased binarized shortFCD areas in the left occipital gyrus, bilateral thalamus, right paracentral lobule, bilateral precentral gyrus, and bilateral postcentral gyrus (Table 1, Figure 4B). Compared with RW, acute SD had significant increased binarized longFCD areas in the right precentral gyrus, bilateral postcentral gyrus, and right superior parietal lobule in the visuospatial network, and decreased binarized longFCD areas in the right supramarginal gyrus in the default mode network (Table 2, Figure 5).

**Table 1:** The binarized shortFCD differences between SD and RW.

**Brain regions of peak coordinates**	**R/L**	**BA**	**Voxel size**	***t*-score of peak voxel**	**MNI coordinates**
					**X, Y, Z**
Cerebellum Posterior Lobe	L	N/A	75	−4.7451	−30 −69 −27
Cerebellum Anterior Lobe	R	N/A	62	−3.8821	51 −48 −36
Cerebellum Posterior Lobe	L	N/A	54	−4.7238	−3 −75 −18
Inferior Frontal Gyrus	R	11	87	−4.0611	18 24 −15
Lingual Gyrus, Middle Occipital Gyrus	L	18, 19	216	5.3825	−15 −93 −12
Thalamus	L, R	N/A	82	5.7899	9 −18 9
Precentral Gyrus, Postcentral Gyrus	R	3, 4, 6	157	4.6467	54 −12 57
Postcentral Gyrus	L	2, 3	72	3.5485	−30 −36 45
Postcentral Gyrus, Paracentral Lobule	R	3, 4	200	4.5221	9 −33 75
Precentral Gyrus	L	6	93	4.2005	−24 −6 63
Postcentral Gyrus	L	3, 5	68	4.7094	−18 −42 57

*Between-group differences in binarized shortFCD thresholded at r = 0.3. The statistical threshold was set at corrected significance level of individual two-tailed voxel-wise p < 0.05 using an AlphaSim corrected threshold of cluster p < 0.05*.

*shortFCD, short-range functional connectivity density; SD, sleep deprivation; RW, rested wakefulness; R, right; L, left; BA, Brodmann's area; MNI, Montreal Neurological Institute; N/A, Not applicable*.

**Table 2:** The binarized longFCD differences between SD and RW.

**Brain regions of peak coordinates**	**R/L**	**BA**	**Voxel size**	**t-score of peak voxel**	**MNI coordinates**
					**X, Y, Z**
Postcentral Gyrus, Precentral Gyrus	R	3, 4	108	5.1923	51 −24 51
Superior Parietal Lobule	R	7, 40	52	3.8173	33 −42 42
Postcentral Gyrus	L	3, 4	40	4.0239	−42 −27 63
Supramarginal Gyrus	R	39	42	−3.6057	51 −66 27

*Between-group differences in binarized shortFCD thresholded at r = 0.3. The statistical threshold was set at corrected significance level of individual two-tailed voxel-wise p < 0.05 using an AlphaSim corrected threshold of cluster p < 0.05*.

*longFCD, long-range functional connectivity density; R, right; L, left; BA, Brodmann's area; MNI, Montreal Neurological Institute; N/A, Not applicable*.

### ROC curve

**Figure 6:**
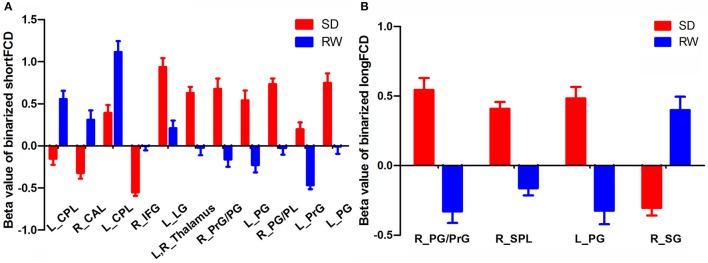
Binarized shortFCD value **(A)** and longFCD value **(B)** of between-group differences in brain areas. Data are mean ± standard error values. L, left; R, right; shortFCD, short-range functional connectivity density; longFCD, long-range functional connectivity density; CPL, Cerebellum posterior lobe; CAL, Cerebellum anterior lobe; IFG, Inferior frontal gyrus; LG, Lingual gyrus; PrG, precentral gyrus; PG, Postcentral gyrus; PL, Paracentral lobule; SPL, Superior parietal lobule; SG, Supramarginal gyrus; SD, Sleep deprivation; RW, Rested wakefulness.

**Figure 7:**
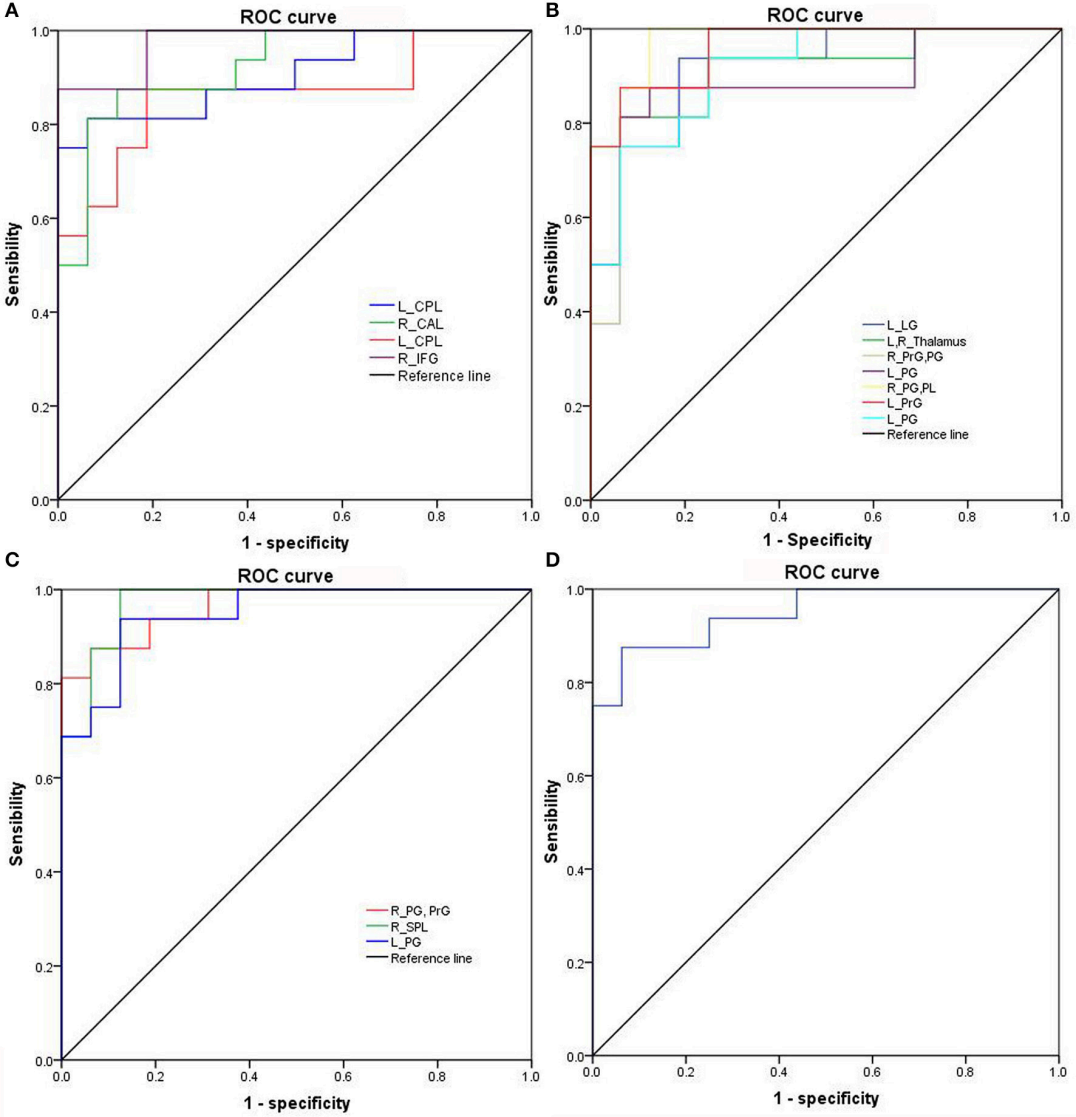
ROC curve of binarized FCD differences in regional brain areas. ROC curve of regional brain areas with decreased binarized shortFCD **(A)**, increased binarized shortFCD **(B)**, increased binarized longFCD **(C)**, and decreased binarized longFCD **(D)**. ROC, Receiver operating characteristic; R, right; L, left; CPL, Cerebellum posterior lobe; CAL, Cerebellum anterior lobe; IFG, Inferior frontal gyrus; LG, Lingual gyrus; PrG, precentral gyrus; PG, Postcentral gyrus; PL, Paracentral lobule; SPL, Superior parietal lobule; SG, Supramarginal gyrus; SD, Sleep deprivation; RW, Rested wakefulness; shortFCD, short-range functional connectivity density; longFCD, long-range functional connectivity density.

The mean beta value of binarized shortFCD (Figure 6A) and binarized longFCD (Figure 6B) differences in those altered brain areas were extracted. These different binarized FCD differences in brain areas were further used for the ROC curve to evaluate their ability to distinguish the acute SD status from the RW status. The area under the curve (AUC) values of those specific binarized FCD differences in brain areas were (mean ± std, 0.933 ± 0.035; 0.863~0.977). Further ROC curve demonstrated that the binarized FCD differences in those regional brain areas alone discriminated the acute SD status from the RW status with high degree of sensitivities (mean ± std, 89.19 ± 6%; 81.3~100%) and specificities (mean ± std, 89.15 ± 6.87%; 75~100%) (Tables 3–4, Figure 7).

**Table 3:** ROC curve for the binarized shortFCD differences in brain areas between SD and RW.

**Brain area**	**AUC**	**Sensitivity (%)**	**Specificity (%)**	Cut off Point^*^
L _Cerebellum Posterior Lobe	0.906	81.3	93.7	0.242
R_Cerebellum Anterior Lobe	0.922	87.5	87.5	−0.0365
L_Cerebellum Posterior Lobe	0.863	87.5	81.2	0.5555
R_Inferior Frontal Gyrus	0.977	87.5	100	−0.2065
L_Lingual Gyrus, Middle Occipital Gyrus	0.91	93.8	81.2	0.3715
L, R_Thalamus	0.922	81.3	93.7	0.5005
R_Precentral Gyrus, Postcentral Gyrus	0.922	87.5	87.5	0.16
L_Postcentral Gyrus	0.887	87.5	87.5	0.006
R_Postcentral Gyrus, Paracentral Lobule	0.977	100	87.5	0.2995
L_Precentral Gyrus	0.961	87.5	93.7	−0.166
L_Postcentral Gyrus	0.914	93.8	75	0.1635

* *Cut off point of mean shortFCD signal value*.

*ROC, Receiver operating characteristic; shortFCD, short-range functional connectivity density; SD, Sleep deprivation; RW, Rested wakefulness; AUC, Area under the curve; R, Right; L, Left*.

**Table 4:** ROC curve for the binarized longFCD differences in brain areas between SD and RW.

**Brain area**	**AUC**	**Sensitivity (%)**	**Specificity (%)**	Cut off Point^*^
R_Postcentral Gyrus, Precentral Gyrus	0.965	81.3	100	0.2845
R_Superior Parietal Lobule	0.973	100	87.5	0.0165
L_Postcentral Gyrus	0.949	93.8	87.5	0.1275
R_Supramarginal Gyrus	0.949	87.5	93.7	−0.048

* *Cut off point of mean longFCD signal value*.

*ROC, Receiver operating characteristic; longFCD, long-range functional connectivity density; SD, Sleep deprivation; RW, Rested wakefulness; AUC, Area under the curve; R, Right; L, Left*.

### Pearson correlation analysis

**Figure 8:**
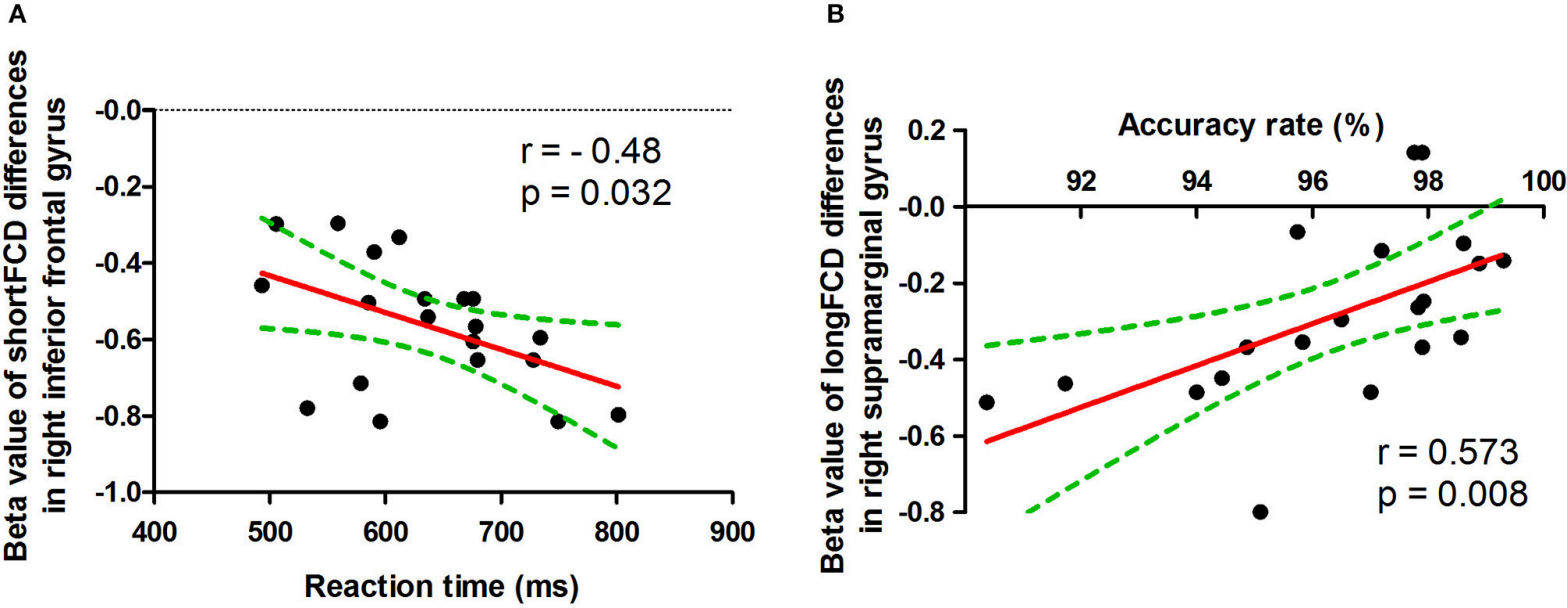
Pearson correlation between beta value of FCD differences in brain areas and ANT. ShortFCD, short-range functional connectivity density; longFCD, long-range functional connectivity density.

The reaction time showed negative correlation with the mean beta value of binarized shortFCD in the right inferior frontal gyrus (*r* = −0.48, *p* = 0.032; Figure 8A), and the accuracy rate demonstrated a positive correlation with the mean beta value of binarized longFCD in the right supramarginal gyrus (*r* = 0.573, *p* = 0.008; Figure 8B). None of the other correlations between the mean beta value of binarized FCD in other different areas and the ANT during the acute SD status were found (*p* > 0.05).

## Discussion

In the present study, we utilized shortFCD and longFCD analysis to characterize the differences of intrinsic functional connectivity induced by acute SD, and their correlations with the ANT. Specifically, we found that acute SD was associated with binarized shortFCD alterations in more regional brain areas than that of binarized longFCD. Acute SD was associated with a significant decrease in binarized shortFCD areas in the cerebellum posterior/anterior lobe and orbitofrontal cortex, and significant increase in the occipital gyrus, thalamus, paracentral lobule, and precentral/postcentral gyrus. Using the binarized longFCD method, only the supramarginal gyrus in the default mode network with decreased binarized longFCD were observed after acute SD relative to RW, and significantly increased binarized longFCD in the precentral/postcentral gyrus and visuospatial network were found. Furthermore, the ANT showed correlations with the beta value of FCD differences in those brain areas during the SD status. Recently, the ROC curve was widely used to applied into the exploration of the reliability of one neuroimaging approach as a potential indicator in distinguishing one group from the other group (1, 40, 41). In general, an AUC value between 0.9 and 1 is considered as excellent, while a value between 0.8 and 0.9 is considered as good. In the present study, the ROC curve demonstrated that the AUC values of the binarized FCD differences in those brain areas showed good discriminating abilities with extremely high AUC values (0.933 ± 0.035; 0.863~0.977). Further diagnostic analysis revealed that the binarized FCD differences in those regional brain areas alone discriminated the acute SD status from the RW status with extremely high degree of sensitivity (89.19 ± 6%; 81.3~100%) and specificities (89.15 ± 6.87%; 75~100%).

The default-mode network is thought to be associated with self-referential mental activity (42), extraction of episodic memory (43), sleep and daydreaming (1, 44), and social cognitive processes related to decision making and self-regulation (45, 46). The orbitofrontal cortex, connected with prefrontal, and deep structures known to mediate sensorimotor processing, motivation, and self-evaluation, is thought to be responsible for mediating the interactions between emotional processes and cognitive functions (47, 48), and play a significant role in fatigue, executive functions, attention, and motivation (49–51). This area is particularly vulnerable to subjects with sleep loss (40, 41, 52, 53). The decreased gray matter volume in the orbitofrontal cortex has previously been reported in patients with daytime sleepiness and chronic insomnia (54, 55). In the present study, we found that acute SD was associated with a significant decreased binarized longFCD within the default mode network node and decreased binarized shortFCD in the right orbitofrontal cortex, which showed an extremely high degree of sensitivity and specificity in distinguishing the acute SD status from the RW status. In addition, the accuracy rate of the ANT demonstrated a positive correlation with the mean beta value of binarized longFCD in the default mode network node, and the reaction time of the ANT showed negative correlation with the mean beta value of binarized shortFCD in the orbitofrontal cortex. We speculated that the decreased long-/shortFCD in the default mode network and orbitofrontal cortex implicated the brain's exertion of voluntary control to remain awake and perform, which might be sensitive biomarkers for advanced cognitive function.

Higher level visual brain areas are divided into two distinct visual pathways: the object properties processing pathway and the spatial properties processing pathway (56–58). The spatial properties processing pathway runs from the occipital lobe up to the posterior parietal lobe and has been called the dorsal system. This system processes object localization and spatial attributes, and is also essential for guiding movements. Damage to the dorsal pathway disrupts the ability to visualize locations or perceive space. The postcentral gyrus is the main receptive region for external stimuli as the location of the primary somatosensory cortex. Recently the postcentral gyrus was implicated with the default mode network (59), which are functional brain hubs showing coupled slow signal fluctuations in the absence of external stimuli during restful waking and sleep (60). The thalamus is a vital region in integrating neural activity from widespread neocortical inputs and outputs, and is thought to play an important role in regulating state of sleep and wakefulness. Previous PET studies have revealed that SD could increase the metabolic rate of glucose in the visual cortex, somatosensory cortex, and fusiform gyrus, which were much higher after 48 h and 72 h than after 24 h SD (61, 62). Previous neuroimaging studies observed disturbed regional spontaneous neural activities in brain areas of the two visual pathways in insomnia patients and individuals after SD (6, 15, 25, 40, 63). In the present study we observed acute SD was associated with altered FCD areas in the thalamus and dorsal system, including significant increased binarized shortFCD areas in the occipital gyrus, thalamus and postcentral gyrus, and increased binarized longFCD areas in the postcentral gyrus and visuospatial network. The increased FCD in these regions in the visual pathway could be considered a compensatory effect to sustain the cognitive performance despite a continuing decline of activity in the higher cognition related areas. This may generate an excessive hyperarousal status, which leads to increased sensory information processing (64).

There are extensive round-trip nerve interactive fibers between the cerebellum posterior lobe(s) and the cerebral cortex. The cerebellum posterior lobe(s) has been widely used for adjusting nerve function, adjusting the start, and planning and coordinating movement. It also works together with the cerebrum to complete functions; such as cognition, language, and emotion; and to initiate, plan, and coordinate movement (65–67). In light of mounting evidence for cerebellar involvement in various neurologic and psychiatric conditions, including obstructive sleep apnea (53), depression (68), primary insomnia (40, 63), mood disorders (69) and sleep deprivation (6); this is crucial. In the present study we found acute SD showed decreased shortFCD in the cerebellum, which may indicate functional deficits associated with decreased ability in adjusting coordinate movement.

## Conclusions

In summary, the longFCD and shortFCD analysis might be sensitive biomarkers to locate the underlying altered intrinsic brain functional organization in individuals during SD status relative to RW status with high degree of sensitivities and specificities. Specifically, the shortFCD analysis is more sensitive to locating the functional organization with more alterations in regional brain areas than that of longFCD. In the present study, we found that the longFCD and short FCDs in the high-order cognition related areas decreased while the arousal and sensorimotor related areas increased to sustain the cognitive performance. These findings expand our knowledge and may help give us insight into a deeper understanding of the neurobiological mechanisms of how the functional organization was altered in the sleep-deprived brain.

There are several limitations that should be noted. First, our study has a relatively small sample size and future studies with a larger sample size is necessary to corroborate our findings. Second, the electronystagmogram has not been used to dynamically monitor the sleep in the SD procedure.

## Author contributions

DK wrote the main manuscript text, DK, RL, JiyZ, and WC conceived and designed the whole experiment, DK, LS, and JiaZ collected the data, DK, RL, and JiyZ analyzed the data.

### Conflict of interest statement

The authors declare that the research was conducted in the absence of any commercial or financial relationships that could be construed as a potential conflict of interest.
